# Probiotic supplementation in marathonists and its impact on lymphocyte population and function after a marathon: a randomized placebo-controlled double-blind study

**DOI:** 10.1038/s41598-020-75464-0

**Published:** 2020-11-02

**Authors:** Helena Batatinha, Edgar Tavares-Silva, Geovana S. F. Leite, Ayane S. Resende, José A. T. Albuquerque, Christina Arslanian, Ricardo A. Fock, Antônio H. Lancha, Fabio S. Lira, Karsten Krüger, Ronaldo Thomatieli-Santos, José C. Rosa-Neto

**Affiliations:** 1 Immunometabolism Research Group, Institute of Biomedical Sciences, University of São Paulo (USP), São Paulo, SP Brazil; 2 Programa de pós-graduação em psicobiologia, Universidade Federal de São Paulo, Santos, Brazil; 3 Laboratory of Applied Nutrition and Metabolism, School of Physical Education and Sports, University of São Paulo, São Paulo, Brazil; 4 Department of Immunology, Institute of Biomedical Sciences, University of São Paulo, São Paulo, Brazil; 5 Department of Clinical and Toxicological Analysis, School of Pharmaceutical Sciences, University of São Paulo, São Paulo, Brazil; 6 Exercise and Immunometabolism Research Group, Post-Graduation Program in Movement Sciences, Department of Physical Education, Universidade Estadual Paulista (UNESP), School of Technology and Sciences, Presidente Prudente, Brazil; 7 Department of Exercise Physiology and Sports Therapy, Justus-Liebig-University Giessen, Giessen, Germany; 8 Department of Bioscience, Universidade Federal de São Paulo, Santos, Brazil; 9 Department of Cell and Developmental Biology, University of São Paulo,, 1524, Prof Lineu Prestes Av., Sao Paulo, SP 05508-000 Brazil

**Keywords:** Immunology, Adaptive immunity, Cytokines, Respiratory signs and symptoms

## Abstract

Probiotic supplementation arises as playing an immune-stimulatory role. High-intensity and -volume exercise can inhibit immune cell function, which threatens athletic performance and recovery. We hypothesized that 30 days of probiotic supplementation could stabilize the immune system of athletes preventing immune suppression after a marathon race. Twenty-seven male marathonists were double-blinded randomly into probiotic (*Bifidobacterium-animalis-subsp.-Lactis* (10 × 10^9^) and *Lactobacillus-Acidophilus* (10 × 10^9^) + 5 g of maltodextrin) and placebo (5 g of maltodextrin) group. They received 30 sachets and supplemented 1 portion/day during 30 days before the race. Blood were collected 30 days before (rest), 1 day before (pre), 1 h after (post) and 5 days after the race (recovery). Both chronic and acute exercise modulated a different T lymphocyte population (CD3^+^CD4^−^CD8^−^ T-cells), increasing pre-race, decreasing post and returning to rest values at the recovery. The total number of CD8 T cell and the memory subsets statistically decreased only in the placebo group post-race. Pro-inflammatory cytokine production by stimulated lymphocytes decreased in the probiotic group after the supplementation period. 30 days of probiotic supplementation maintained CD8 T cell and effector memory cell population and played an immunomodulatory role in stimulated lymphocytes. Both, training and marathon modulated a non-classical lymphocyte population regardless of probiotic supplementation.

## Introduction

Over the last few years, various studies have shown a close interaction between the microbiota and the peripheral immune system^1,2^. Gut microbiota is composed by billions of different species of bacteria with pathogenic or symbiotic characteristics, depending on the environment homeostasis. This microbiota performs many immunoregulatory functions and plays a role in immune development and function^3–5^. In this field, probiotic supplementation arises as a strategy to treat metabolic, autoimmune and inflammatory diseases^6^. Probiotics are classified as commensal bacteria living in a symbiotic manner with the host^7^. They compete for nutrient with pathogenic bacteria, preventing their colonization. Probiotics also have an immune-stimulatory effect. Milk enriched with *Bifidobacterium lactis* (HN019 5 × 10^9^) increased the number of neutrophils and CD4 and CD8 T lymphocytes^8^. In addition, 11 weeks of *Lactobacillus fermentum* (1 × 10^9^ UFC) supplementation prevented upper respiratory tract infections (URTI) in endurance athletes^9^.

High-intensity and -volume exercise such as a marathon race has been associated with immunosuppression by decreasing immune cell function, which enhances susceptibility to viral infections^10,11^. After a marathon race, the function of neutrophils, T lymphocytes and Natural-killer (NK) cells, as well as salivary immunoglobulin A (IgA), are reduced^12^. Moreover, a single bout of exhaustive exercise increases cortisol concentration in the plasma. High levels of cortisol inhibit lymphocyte proliferation and monocyte function^13^. Similarly, decrease in energetic substrates for immune cells, such as glucose and glutamine, has been found^14^. All these events might contribute to increased susceptibility to URTI.

Consequently, several studies started to investigate strategies to avoid immunosuppressive periods and prevent the incidence of URTI, e.g. acute or chronic supplementation of glucose and glutamine^15–17^. The results showed that a single bout of exhaustive exercise by itself is not able to increase susceptibility to URTI unless the athlete is in overtraining or fatigue condition. Thus, boosting the immune system of athletes during training is more effective than acute intervention before a race.

Regarding these points, we hypothesized that 30 days of probiotic supplementation would strengthen the immune system of athletes and prevent a decrease in immune function after a marathon race. We aimed to evaluate the alterations caused by a marathon in the lymphocyte population and function, and the effects of probiotics in this process.

## Results

The anthropometry and physical characteristics of the athletes are shown in Table 1. Probiotic group showed higher fat mass percentage and body density comparing to placebo. Data from the questionnaires showed no disease, medicine or immune boosting supplements consumption by any athlete and an average sleep of 6 h/night in both groups (data not shown). The average race time was 4.08 ± 0.55 h. Non-statistical difference was observed for the training volume during the 30 days of supplementation (Placebo 3.93 ± 1.73 h/week, 62.83 ± 49.79 km/week; Probiotic 5.03 ± 2.63 h/week, 56.06 ± 25,59 km/week) (Supplementary Table 1.

**Table 1:** Anthropometry and physical characteristics of the athletes.

	Placebo (n = 13)	Probiotic (n = 14)	*p*
Age (years)	40.46 ± 7.79	35.96 ± 5.81	0.09
High (m)	1.75 ± 0.08	1.75 ± 0.06	0.78
Weight (kg)	72.67 ± 10.20	79.30 ± 10.99	0.11
Fat mass (%)	11.32 ± 4.40	16.9 ± 5.80*	0.01
Free fat mass (%)	61.12 ± 9.03	62.51 ± 6.78	0.89
Body density (kg/l)	1.06 ± 0.01	1.05 ± 0.01*	0.02

Data presented as mean ± standard deviation, *Different from placebo. Significant when *p* < 0.05.

### Cytokine production in whole blood under LPS stimulation

**Figure 1:**
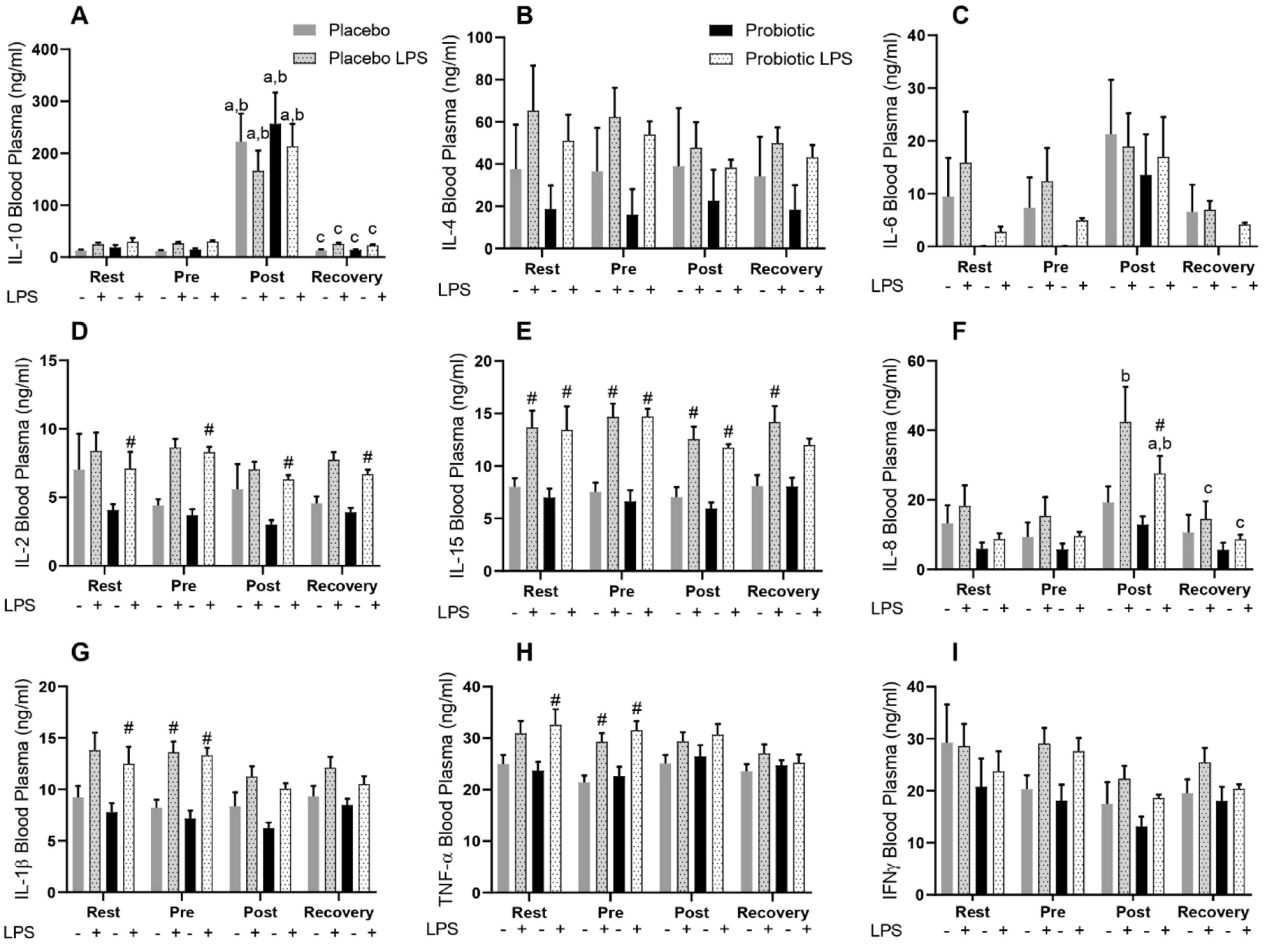
Concentrations of cytokine in plasma stimulated or not with LPS, IL-10 (**A**), IL-4 (**B**), IL-6 (**C**), IL-2 (**D**), IL-15 (**E**), IL-8 (**F**), IL-1β (**G**), TNFα (**H**) and IFNγ (**I**), of Marathonists supplemented with placebo (grey bar) or probiotic (black bar). ^a^Different from Rest. ^b^Different from Pre. ^c^Different from Post. ^#^Different from LPS in the same group (two-way ANOVA followed by Tukey). (N = 12 in each group).

Interleukin (IL)-10 increased in both groups regardless of lipopolysaccharides (LPS) stimulation post-race (Fig. 1A), suggesting an effect of exercise. Non-statistical difference was observed in the levels of IL-4 (Fig. 1B) and IL-6. However, at the times rest, pre-race and recovery the probiotic group produced IL-6 only with stimulation (Fig. 1C). In both groups, a similar increase on IL-15 and IL-2 production under LPS stimulation was found (Fig. 1D and E). However, the change of IL-2 was only statistically significant in the probiotic group. IL-8 was responsive to LPS only post- race in both groups (Fig. 1F). An impaired LPS-induced IL-1β and Tumor Necrosis Factor alpha (TNFα) was found after the race, which had not been recovered (Fig. 1G and H). No significant changes in the concentration of Interferon gamma (IFNγ) was found (Fig. 1I).

### Probiotic supplementation maintained total CD8 T cells and memory phenotype population after the race

The changes in blood cell numbers are shown in Supplementary Table 2. No effect of probiotic supplementation was found. In both groups, total leucocytes numbers increased post-race. The sharpest increase was found for neutrophils, followed by monocytes, while lymphocyte numbers decreased. All cell populations returned close to the rest levels at recovery.

**Figure 2:**
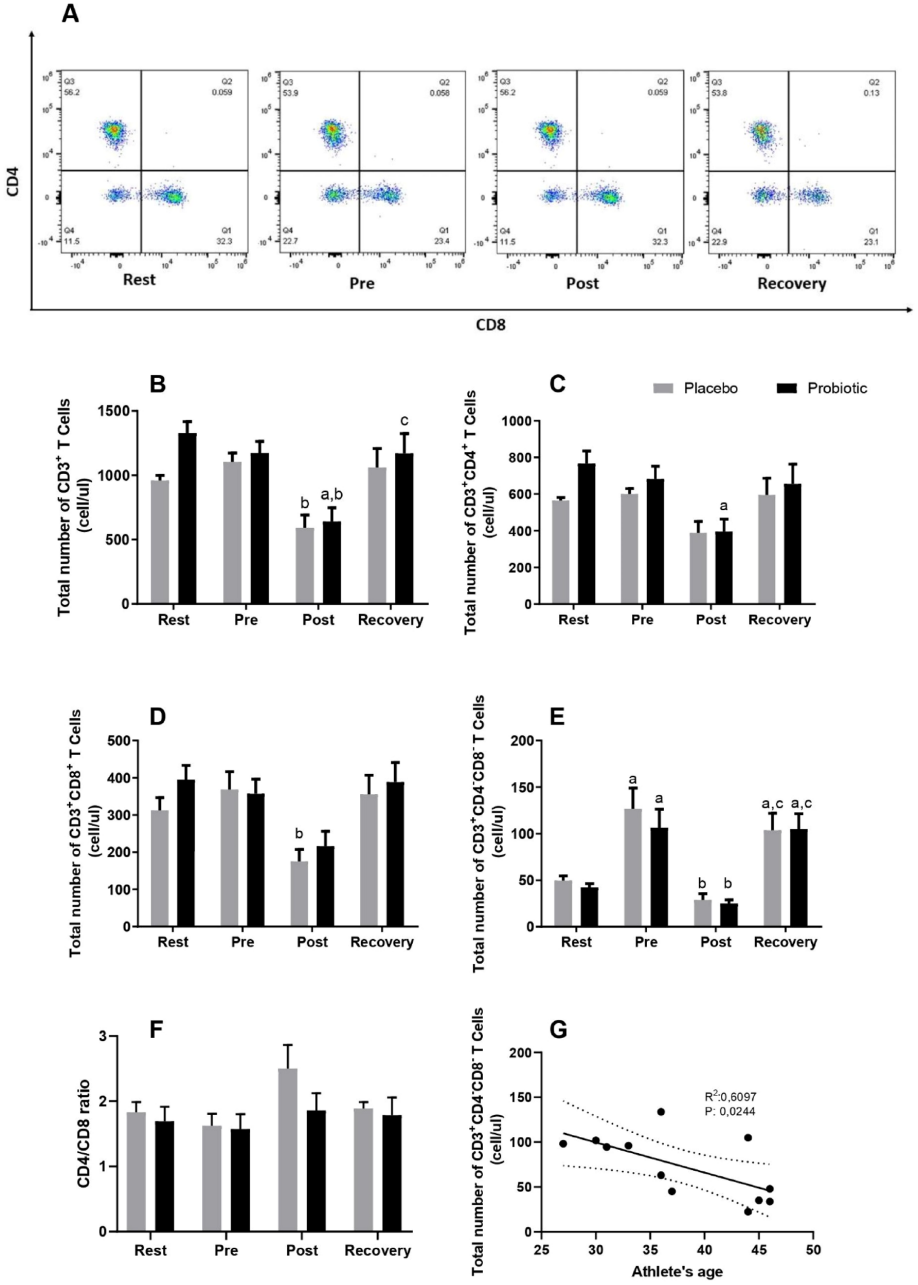
Dot plot representing the percentage of T lymphocyte subpopulations in response to the marathon (**A**) and total number of lymphocyte subsets CD3^+^ (**B**) CD3^+^CD4^+^ (**C**) CD3^+^CD8^+^ (**D**) CD3^+^CD4^−^CD8^−^ (**E**). CD4/CD8 ratio (**F**) and the correlation between CD3^+^CD4^−^CD8^−^ T cells and athletes’ age (**G**). Marathonists supplemented with placebo (grey bar) or probiotic (black bar). ^a^Different from Rest. ^b^Different from Pre. ^c^Different from Post. (Two-way ANOVA followed by Tukey). (N = 12 in each group).

The representative flow dot-plot of lymphocytes showing CD4^+^, CD8^+^ and CD4^−^CD8^−^ T cells percentage is presented on Fig. 2A. The total number of CD3^+^ T cells decreased in both groups after the race; in the probiotic group, it was regulated back to baseline at recovery (Fig. 2B). CD4^+^ T cell decreased post-race compared to rest in the probiotic group (Fig. 2C), while CD8^+^ T cells decreased only in the placebo group in response to the marathon race (*p* < 0.05) (Fig. 2D). No change was observed in the CD4/CD8 ratio between the groups at any time (Fig. 2F).

**Figure 3:**
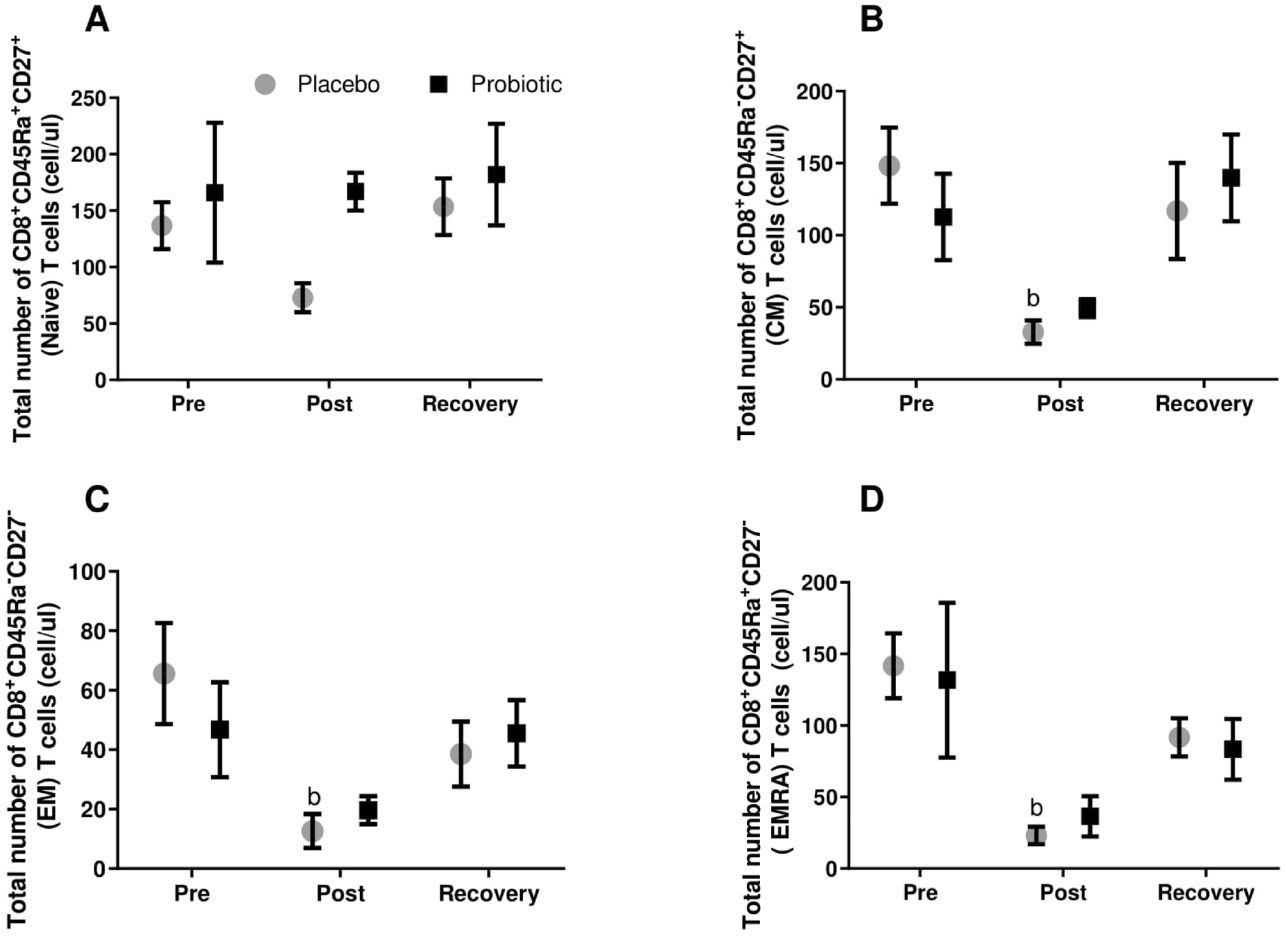
Total number of CD8 T memory subsets: Naïve (**A**); central memory (**B**); effector memory (**C**); effector memory RA (**D**). Marathonists supplemented with placebo (grey circle) or probiotic (black square). ^b^Different from Pre (two-way ANOVA followed by Tukey). (N = 6 in each group).

In order to understand if the race could impact immunological memory phenotype and if probiotic supplementation could modify it, memory phenotype of CD4^+^ and CD8^+^ T cells was analyzed. For CD8^+^ T cells, the total counts of effector memory (EM), central memory (CM) and effector memory expressing CD45Ra (EMRA) statistically decreased only in the placebo group in response to the marathon. Numbers of naïve CD8^+^ T cells were twice as high in the probiotic group after the race as in the placebo group; however, the increase was not statistically significant (Fig. 3). For the CD4^+^ T cell population, the probiotic group tended to increase EMRA CD4 T cells at the recovery comparing to placebo group (*p* = 0.06) (Supplementary Fig. 1).

### The marathon race modulated the non-classical double negative T lymphocyte population

Athletes started with approximately 11% of the non-classical double-negative T cell population (at rest), which doubled pre-race—comprised in an intense period of training. These cells decreased post-race compared to pre and increased again at recovery (Fig. 2E).

**Figure 4:**
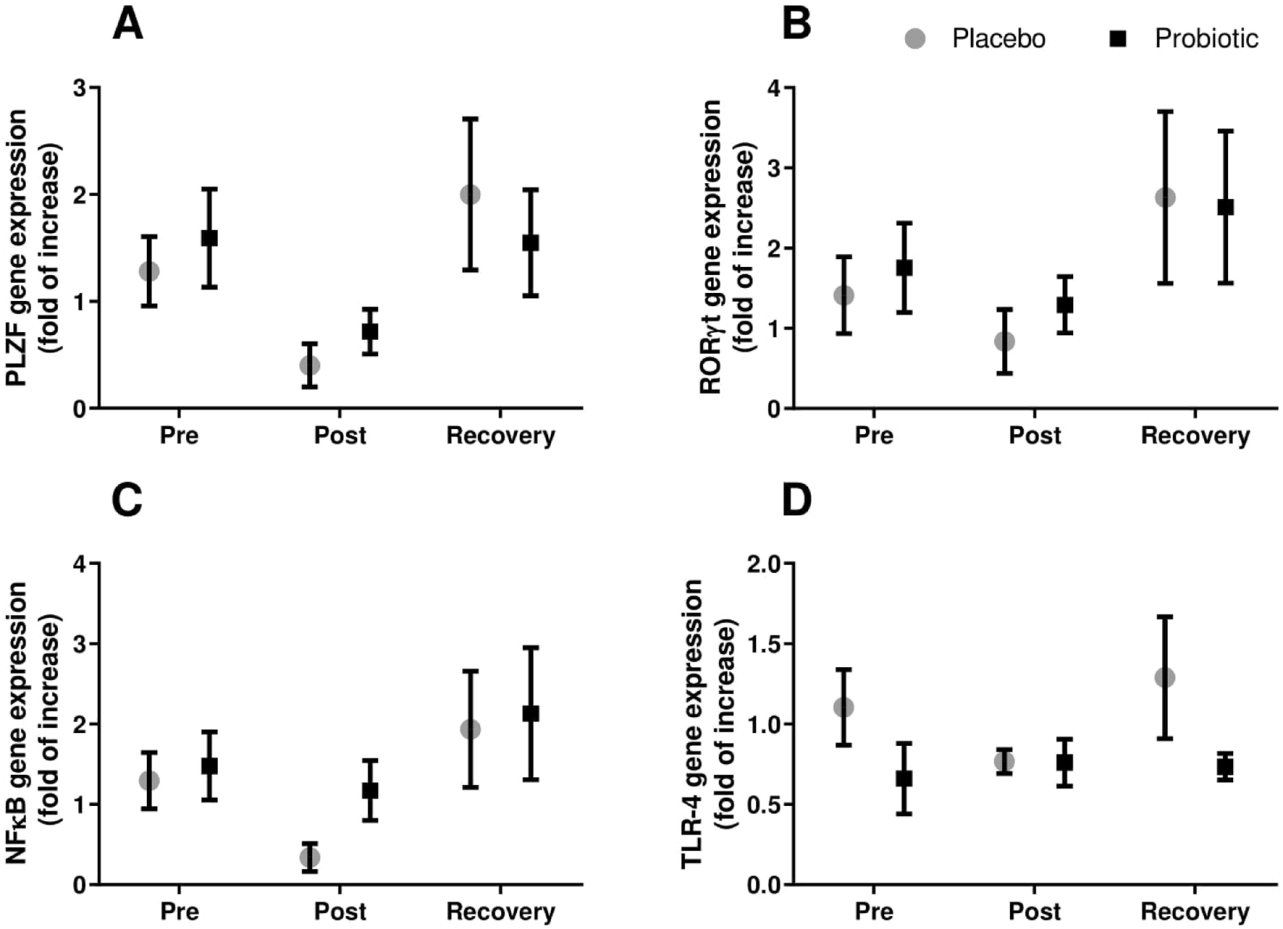
Gene expression of PLZF (**A**), RORγt (**B**), NFκB (**C**) and TLR-4 (**D**) of lymphocytes cultured for 24 h with PMA from marathonists supplemented with placebo (grey circle) or probiotic (black square). (Two-way ANOVA followed by Tukey). (N = 6 in each group).

Moreover, these cells presented a negative correlation with age (Fig. 2G), also observed in Gamma-delta (γδ) T and Mucosal associated invariant T (MAIT) cells (see “Discussion” section). The promyelocytic leukemia zinc finger protein (PLZF) gene expression; a transcription factor expressed in γδ T cells and MAIT cells, showed a response to the marathon similar to the double-negative T cells (Fig. 4A). RORγt mRNA followed the same pattern (Fig. 4B).

### Probiotic supplementation induces immunomodulation in stimulated lymphocytes.

**Figure 5:**
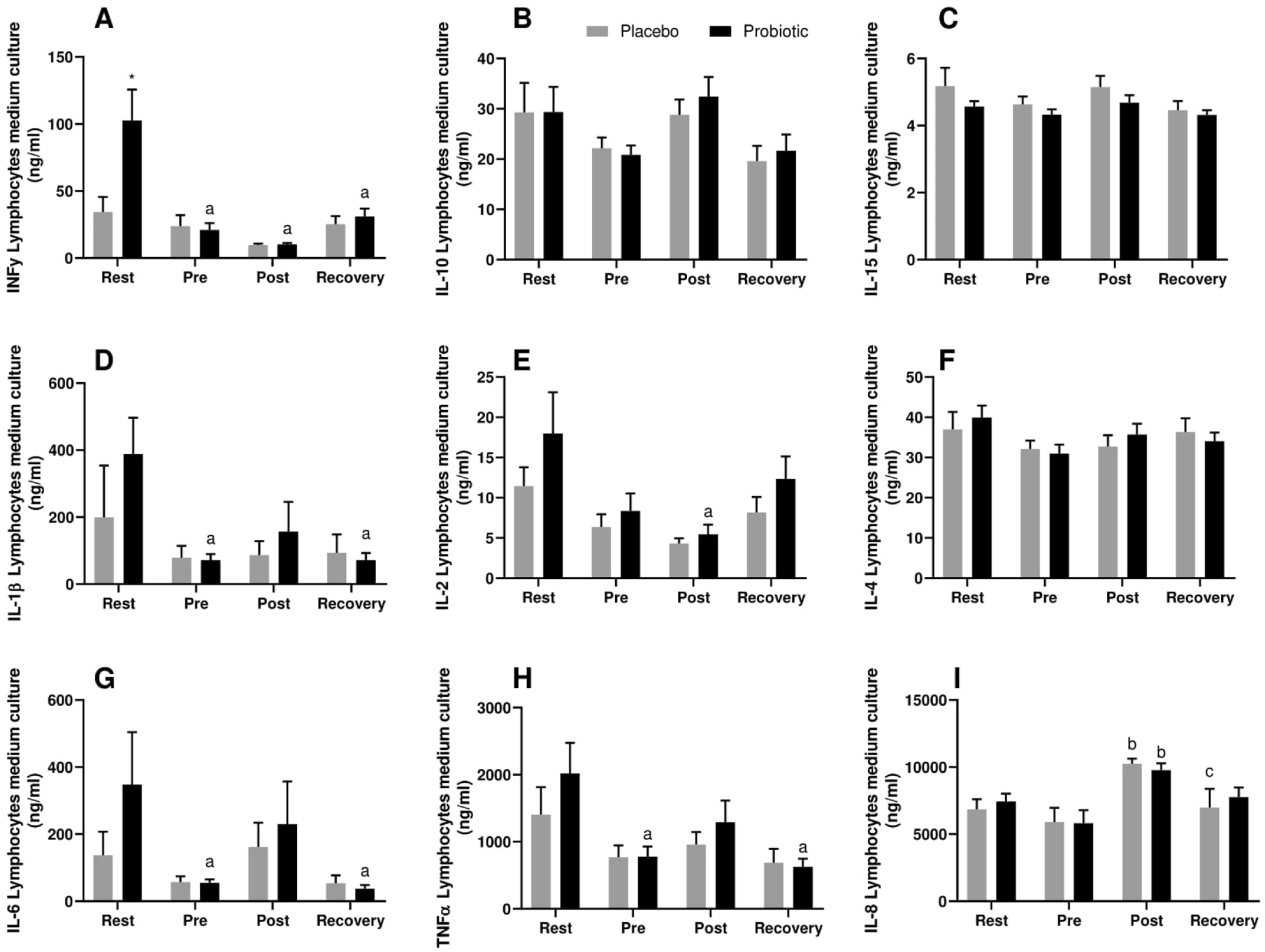
Cytokine production by lymphocytes in the culture medium stimulated with PMA. IFNγ (**A**), IL-10 (**B**), IL-15 (**C**), IL-1β (**D**), IL-2 (**E**), IL-4 (**F**), IL-6 (**G**), TNFα (**H**), IL-8 (**I**) in Marathonists supplemented with placebo (grey bar) or probiotic (black bar). ^a^Different from Rest. ^b^Different from Pre. ^c^Different from Post. (Two-way ANOVA followed by Tukey). (N = 10 in each group).

Stimulated lymphocytes from the probiotic group, presented a two-fold increase of pro-inflammatory cytokines production (IFNγ, IL-1β, IL-6 and TNFα) at rest compared to the placebo (only for IFNγ *p* < 0.05) (Fig. 5). After 30 days of probiotic supplementation (Pre-race), all pro-inflammatory cytokines significantly decreased comparing to rest (Fig. 5A,D,G and H); IL-2 decreased post-race compared to rest in the probiotic group, whereas it was partially restored at recovery (Fig. 5E). IL-8 increased in both groups post-race compared to pre-race and decreased only in the placebo group at recovery (Fig. 5I). Non-statistical significance was observed for IL-10, IL-15 and IL-4 (Fig. 5B,C and F). Similarly, no significant changes were found for gene expression of the Nuclear factor kappa B (NFκB) and Toll-like receptor 4 (TLR-4) (Fig. 4). IL-17 was not detected to any group in any time point.

### Probiotic supplementation did not prevented URTI

**Figure 6:**
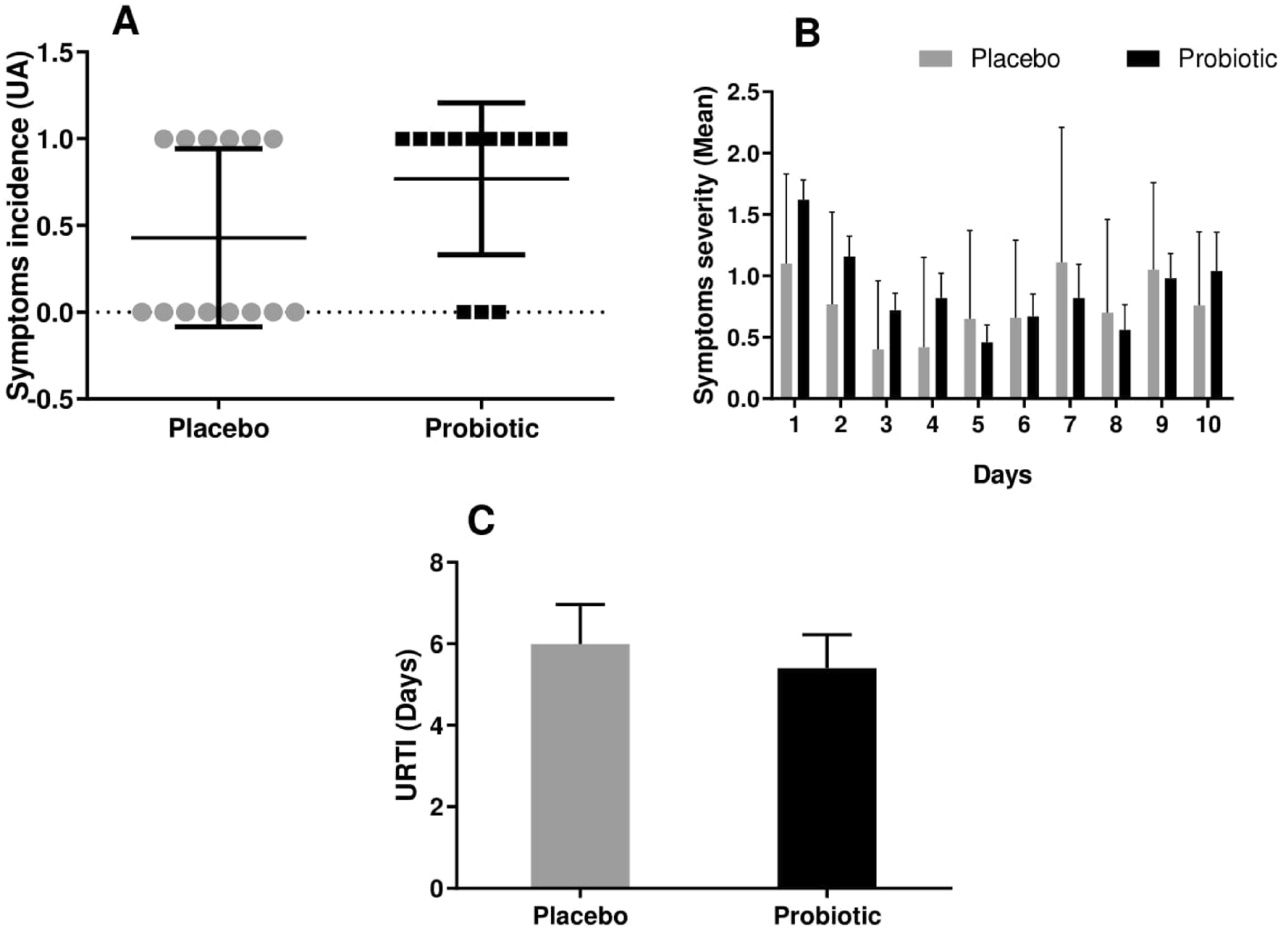
Symptoms incidence (**A**), symptoms severity (**B**) and upper respiratory tract infection (**C**) reported by marathonists supplemented with placebo (grey bar) or probiotic (black bar). during 10 days after the race. (Two-way ANOVA followed by Tukey). (N = 13 in each group).

Data from the URTI questionnaire showed that both placebo and probiotic group presented low level of URTI with no statistical difference between the groups. In addition, both groups presented at least one upper respiratory symptom during ten-days after a marathon race. However, the symptoms reported were of low severity level in both groups with non-statistical difference been observed between the groups regarding the incidence and severity score of symptoms during this ten-day period (Fig. 6).

## Discussion

One bout of high-intensity and prolonged exercise is known to induce acute leukocytosis and increase concentrations of cytokines such as IL-6 and IL-10 in the serum. Our findings reproduced this classical immunologic response and 30 days of probiotic supplementation did not affect this response. However, the selective characterization of immune cell subtypes added information about the kinetics of changes of CD3^+^CD4^−^CD8^−^ T cells. These cells increased proportionally with the training period (rest to pre-race) regardless probiotic supplementation, decreased in response to the marathon (post) and were regulated to pre-race levels five days after (recovery). The cells are seldom investigated in exercise immunology and, as we will discuss below, it may be due to the differential regulation of γδ T cells and/or MAIT cells, which represent two important T cell subtypes of mucosal immunity. Probiotic supplementation affected total number of CD8^+^ T cell and memory phenotype; and regulated lymphocytes response to stimulation.

The increase in circulating immune cells in response to high-intensity and prolonged exercise, such as a marathon race, was previously described by Davidson and collaborators (1987)^18^. It happens in a catecholamine-dependent manner, and cells tend to return to baseline values within few hours after exercise cessation^19^. Neutrophils are the cell type most commonly responsible for this increase, with a small contribution of monocytes and NK cells, while lymphocyte numbers are temporarily decreased^20^. Cytokine concentration in serum is also altered in response to acute exercise. IL-6, known as a pleiotropic cytokine, is sharply increased in circulation in response to muscle contraction during exercise. This muscle-derived IL-6 has an important anti-inflammatory effect by inducing IL-10 and IL-1 receptor antagonist (IL-1Ra) production^21^. Our results reproduced these findings, since leukocytosis and an increase in both IL-6 and IL-10 post-race was found regardless of LPS stimulation.

In order to characterize the lymphocyte subpopulation, a whole blood immunophenotyping was performed. There was a significant decrease in the amount of CD3^+^ T cells in both groups and of CD8^+^ T cells in the placebo group after the race. It presented a profile similar to the total lymphocyte number; the amount of CD4^+^ T cells was not altered. Tossige-Gomes and collaborators (2014)^22^ have not found significant differences in the amount of T helper (Th) (CD3^+^CD4^+^) or T cytotoxic (CD3^+^CD8^+^) cells after an exhaustive exercise session. However, another study indicated a decrease in the percentage of CD8^+^ T cells in response to exhaustive exercise, without changing total cell numbers^23^.

Cytotoxic T cells are important to neutralize viral infections and tumor growth, since they directly inactivate infected and defective cells, preventing its proliferation. Human immunodeficiency virus (HIV) and hepatitis C patients presented decreased number and function of CD8^+^ T lymphocytes^24,25^, indicating that a decline in this cell population may contribute to acute infections. As described above, the probiotic group did not present a significant decrease in CD8^+^ T cells one hour after the race.

The memory CD8^+^ T cell subset was also affected by the race and probiotic supplementation. The total number of EM, EMRA and CM decreased post-race only in the placebo group, returning 5 days after. Spielmann and collaborators (2013)^26^ found that 30 min of cycling (performed at 80% VO_2_max) mobilized CD8^+^ naïve cells more effectively in younger individuals compared to older ones. Exercise was also able to mobilize highly differentiated and EMRA CD8^+^ T cells subsets. In another study from the same group^27^, a similar exercise protocol increased both CD4^+^ and CD8^+^ T cells EM and EMRA subsets; this difference between pre- and post-exercise was maintained after eight days of ex-vivo cell expansion. As long as these cells differentiate, they lose their proliferative capacity, however, they react quickly to pathogen stimulation, improving immune response^28^. A decrease in EM, EMRA and CM populations might increase susceptibility to viral infections.

T helper cells produce pro- and anti-inflammatory cytokines that recruit and activate monocytes, B lymphocytes and cytotoxic lymphocytes, modulating immune response according to the stimulus^29^. In our results, we found that at rest, lymphocytes in the probiotic group presented exacerbated production of the pro-inflammatory cytokines IFNγ, IL-1β, IL-6 and TNFα when stimulated. This over-response was restored pre-race. This suggests that 30 days of probiotic supplementation could modulate immune response of lymphocytes to stimulation. IL-2 decreased post-race compared to rest in the probiotic group. However, it was partially restored 5 days after the race (recovery). This cytokine is mainly produced by activated lymphocytes and can be considered as a T cell growth factor by stimulating lymphocyte proliferation, survival and effector function^30^.

Within the lymphocyte subpopulation, the highest modulation by the marathon race was found for CD3^+^CD4^−^CD8^−^ T cells. Representing around 5% of the total circulating T cells; these non-classical lymphocytes have been described in the literature a few years ago and play an important role in responding quickly to viral and bacterial infections. One of the first types described was Invariant Natural Killer (iNKT) cells—they recognize antigens by the molecule CD1d, which is located in the surface of antigen-presented cells^31^. Once activated, iNKTs produce a large amount of IL-4 and IFN-y, also having a cytotoxic effect^32^. MAIT cells are another type of non-classical lymphocytes recently described. When activated, these cells are fast and efficient to release IFNγ, TNF-α, granzyme B, perforins, IL-17, and IL-22^33,34^. MAIT cells are directly correlated with anti-microbial immunity, since they are absent in germ-free animals^35^. It also plays a role in mucosal immunity by preventing respiratory infections^36^. The circulating concentration of these cells are decreased in patients with infectious and inflammatory bowel disease (IBD)^37,38^.

γδ T cells are cytotoxic effector non-classical lymphocytes that play an important role in rapid response to a variety of pathogens, removing bacteria and eradicating tumor cells^39,40^. They are primarily located in the reproductive, respiratory and gastrointestinal epithelium, but also represent 2–15% of the total CD3^+^ circulating cells^40^. γδ T cells are highly responsive to acute stress and immediately increase in the circulation in response to short-term acute exercise. Nevertheless, these cells also seem to be affected by chronic exercise, since they were found to be proportionally higher in trained female soccer players compared to untrained individuals^41^.

Although we have not observed differences between the probiotic and placebo group regarding the CD3^+^CD4^−^CD8^−^ T cell population, we found a significant increase in the amount of non- classical lymphocytes pre-race compared to rest. In this period, athletes were in a high-volume regime of training, indicating that chronic high-intensity and volume exercise may modulate this cell population. Moreover, these cells were also affected by a single bout of exercise, since they decreased one hour after the marathon, which suggests that both chronic and acute exercise may modulate non-classical T lymphocytes.

The transcription factor PLZF is highly expressed and essential for MAIT and γδ T cell effector function^42,43^. PLZF was observed to decrease in patients with Human T-lymphotropic virus type 1 (HTLV-1), a deltaretrovirus infection^44^. RORγt is also expressed by both cell populations and is responsible for their ability to release IL-17^43,45^. Although not significant, we observed a similar path between the gene expression of PLZF and RORγt with the non-classical lymphocytes, decreasing post-race and increasing at recovery.

Furthermore, both MAIT^46^ and γδ T cells^47^—unlike iNKT cells^48^—are reduced in elderly people, which indicates a negative correlation between age and the number of circulating cells. In our results, we found this correlation (see Fig. 5F), which is a strong indicative that these CD3^+^CD4^−^CD8^−^ T cells may be both MAIT and γδ T cells, with more contribution of γδ T cells, since they are affected by acute and chronic exercise. However, we have not labelled these cells with specific antibodies to detect the specific population.

In order to understand if probiotic supplementation affects the incidence of URTI after a marathon, a questionnaire was applied from day zero to ten after the race. Both groups presented low level of URTI symptoms during this period; However, neither the placebo nor the probiotic group showed significant symptom severity or incidence during these ten days. In previous studies^49^, the “open window” hypothesis was discussed. This concept describes that up to 72 h after intense and prolonged exercise, the immune cells exhibit poor response against pathogens, which increase susceptibility to infection. It was speculated that this acute immunosuppression was the result of a decrease in substrate concentration since it was at least partly prevented by acute glucose and/or glutamine supplementation^50^.

In the last decade, however, scientists have associated increased URTI incidence with repeated bouts of strenuous exercise without proper recovery (overtraining), culminating in chronic immunosuppression^50,51^. In this regard, chronic supplementation of specific substrates might be an effective strategy. We have not evaluated the URTI symptom before the race. It is possible that the low level of URTI was already present before the race, as a consequence of the training volume.

It is likely that the period of supplementation (30 days before the race) was a limitation of the study and could have mitigated possible benefits. West and colleagues (2013)^52^ supplemented physical-active individual with the same probiotic mix that we used, in a lower dose (1 × 10^10^), for 150 days. They found that probiotic group had 27% reduced risk to URTI and were less absent in the training sections than the placebo group. In another study from the same group^53^ with the same supplement dosage and period; probiotic decreased circulating matrix metalloproteinases 1 (MMP-1), a extracellular matrix degrading enzymes that has been implicated in atherogenesis, but had no difference in the activities of Neutrophils, Monocytes and NK cells. They suggest that probiotic supplementation, besides reducing URTI risk, had no effect on cytokines production and cellular activity because the group supplemented was immunocompetent; the results could be different if tested in children, elderly or endurance athletes.

Furthermore, it is important to highlight that both studies were performed in rest conditions, so the effects of a single bout of exercise on the immune cells and the interactions of probiotics in this context were not evaluated. Here we presented data from before and after 30 days of supplementation, after the race, and on the recovery. We found the probiotic supplementation, in rest condition, modulated lymphocytes’ response to stimulation; and after an acute stress (Marathon race) played a role on the CD8^+^ T cells population of marathon runners.

In conclusion, even though we have not found changes in URTI incidence in the athletes, 30 days of probiotic strain supplementation was able to maintain the total number of CD8^+^ T cells and effector memory population; and to modulate lymphocytes’ response to stimulation, suggesting a role of probiotics in the athletes’ immune system. This paper also opened a new avenue in the immunology field, showing that both chronic and acute high-intensity and -volume exercise can modulate an important lymphocyte subpopulation (CD3^+^CD4^−^CD8^−^ T cells). The limitations of this study are the period of supplementation, not having evaluated URTI symptoms before the race and marked the double-negative T cell population; and not having accessed the microbiota composition of the athletes. More studies with different supplementation schedules and probiotic composition are necessary to better understand the impact of probiotics in the immune system of athletes. Further studies are also necessary to identify the role of non-classical T lymphocytes in trained individuals.

## Methods

### Ethical approval

All the experimental protocol of this project was approved by the Human Research Ethics Committee of the Biomedicine Institute of Universidade de São Paulo and by the Brazilian National Research Ethics Committee. It was registered as a clinical trial on January 31st, 2017 by the protocol number: 61892116.9.0000.5467.

All methods were carried out in accordance with relevant guidelines and regulations. Informed consent was obtained for all the participants. They received orientations regarding the protocol and assigned an informed consent statement confirming they were aware of the benefits and risks and they could quit at any time.

### Participants

Forty male marathonists aged between 30–45 years volunteered for the study. The participants were randomized in a double-blind way (utilizing the program MINITAB) by a draw at upon their enrollment in the study. A researcher who was involved with the study was responsible for the randomization and the supplementation delivery. Thirteen participants abandoned the protocol (7 in the placebo and 6 in the probiotic group), whether because they could not run on the day of the race, were unable to finish the race or decided to quit before the end of the study; thus, 27 athletes remained (n = 13 in the placebo group, n = 14 in the probiotic group). The inclusion criterion was having completed at least one marathon before the study, being regular runner for at least 2 years, aged between 25 and 45 years and non-consumers of probiotic products. The exclusion criteria were smoking, any known cardiovascular, metabolic, or neurological disease, consumption of any probiotic product, use of medication that could mistake the results (antibiotic and anti-fungi in the last 6 months previous the first data point; anabolic steroids and hormones), regular use of any supplements but carbohydrates, protein and vitamins (which were all reported) . All volunteers answered a questionnaire informing nutritional intake (dietary record of 24 h), quality of sleep and weekly training volume report (training record).

### Supplementation

Each athlete in the probiotic group received 30 sachets containing *Bifidobacterium animalis. subsp. Lactis* (10 × 10^9^) and *Lactobacillus Acidophilus* (10 × 10^9^) associated with 5 g of maltodextrin (Drogafarma, São Paulo, Brazil). The probiotic stain and the guidelines for consumption were based on Nicholas West’ work^52^. The placebo group received 30 sachets in the same shape, weight and flavor as the probiotic containing 5 g of maltodextrin. All athletes were structed to consume one sachet per day dissolved in water (preferably at night before the sleep) during the thirty days before the race. A researcher from our group contacted all the athletes every 2 days to ask if the sachets were being consumed properly and if they had any complication (gastrointestinal symptoms, lack of sleep, pain or some other discomfort) related to the supplementation. All the athletes consumed the sachets properly and any complication regarding the supplementation was reported.

### Experimental procedure

**Figure 7:**
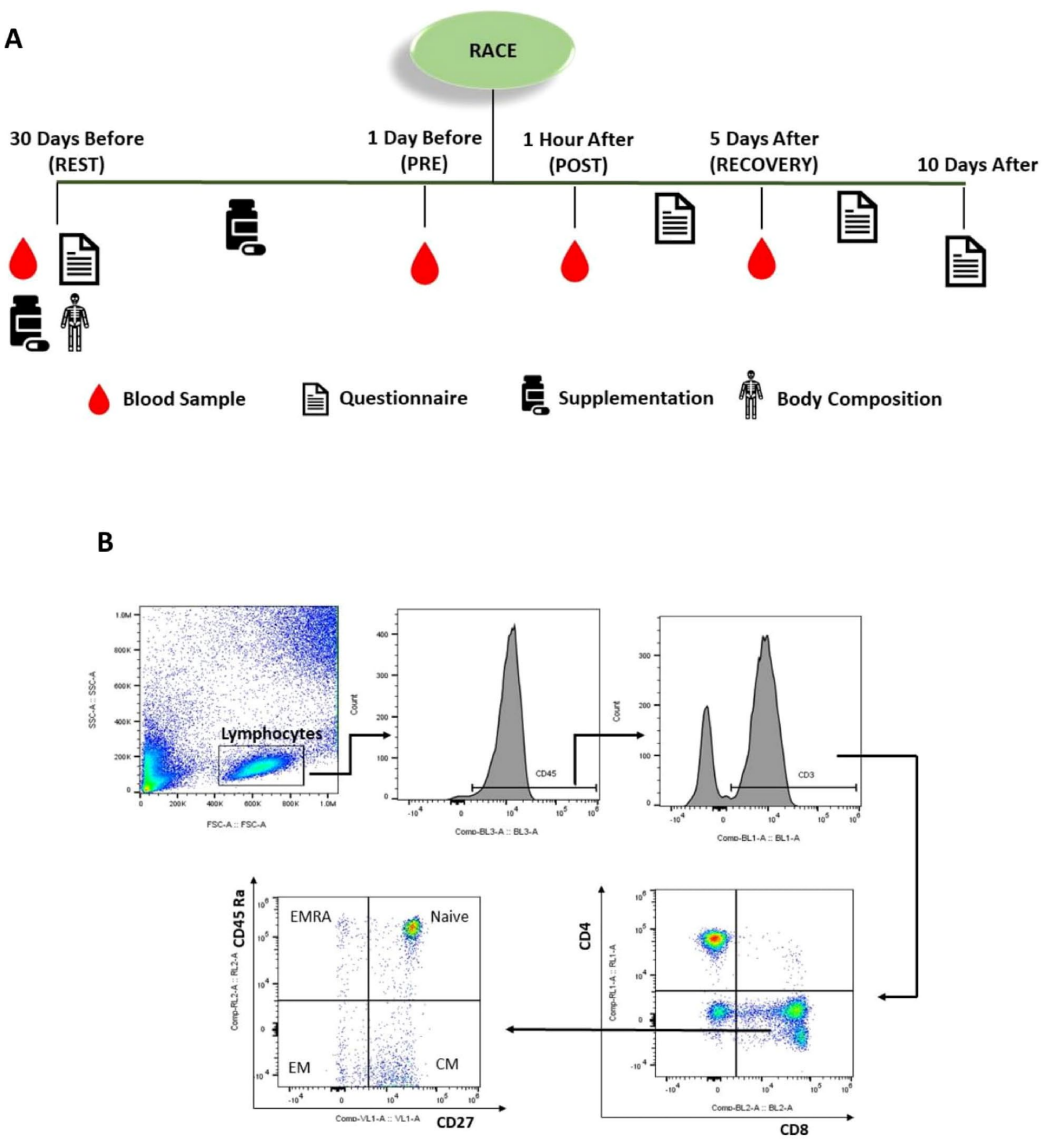
Experimental design (**A**) Gating strategy of lymphocyte populations by flow cytometry (**B**). CD45 (BL-3), CD3 (BL-1), CD4 (RL-1), CD8 (BL-2), CD45RA (RL-2), CD27 (VL-1). CM (central memory) EM (effector memory) EMRA (effector memory RA).

The experimental design is outlined in the Fig. 7A. Athletes were asked to report to the Laboratory of Nutrition and Exercise at the Sports and Physical Education School—University of São Paulo 30 days before the race, where they signed an inform consent, agreeing to participate in the study. They answered the questionnaires and had their body composition analyses drawn by air displacement plethysmography (BOD POD body composition system; Life Measurement Instruments, Concord, CA, USA), and their first blood sample collected (Rest). The Athletes were randomized in a double-blind manner into probiotic and placebo group and received the sachets for supplementation. On the day before the marathon, athletes were asked to return to the laboratory for blood sample collection (Pre). Food and water intake were reported immediately after the race; sensation of fatigue, race pace and the exact time they finished were recorded. The blood sample were collected one hour after the marathon (Post). Athletes were asked to return to the laboratory for the last blood sample collection five days after the marathon (Recovery).

### Upper respiratory tract infection questionnaire

In order to evaluate URTI symptoms, the Wisconsin Upper Respiratory Symptom Survey—21 (WURSS—21), were applied from day zero to day ten after the race. The questionnaire consists of 21 questions investigating health and correlations with flu symptoms. All items are based on the 7-point Likert scale. To verify the installation of URTI two or more symptoms should occur for three or more days in a sequence, same as the protocol utilized by West and colleagues (2013)^52^.

### Blood processing

All blood samples were processed within 4 h after collection. Blood count for total leukocytes, neutrophils, monocytes, lymphocytes and platelets were obtained from EDTA anti- coagulated blood from the laboratory of the University of São Paulo hospital. Whole-blood LPS stimulus was obtained from the EDTA anti-coagulated blood; 2 ml of blood were stimulated with 200 ul of LPS during 1 h at 37 °C, with constant and slow rotation. Immediately after incubation, plasma was collected following the centrifugation procedure (3000 rpm, 15 min, 4 °C) and stored at −80 °C for further analyses. 2 ml of blood without LPS were incubated for the same time and under the same conditions for control. Serum was obtained from dry blood tube by means of centrifugation (3000 rpm, 15 min, 4 °C); half of which was used for cell culture, and the other half, stored at −80 °C for further analysis.

### Lymphocyte isolation and cell culture

Peripheral blood mononuclear cells (PBMCs) were isolated from 5 ml of EDTA anti-coagulated blood using Histopaque-1119 and Histopaque-1077 (Sigma-Aldrich, Merck KGaA, Darmstadt, Germany). The PBMCs were washed with 12 ml of PBS for pellet generation by centrifugation (1800 rpm, 10 min, 4 °C). The PBMC pellet was resuspended in an RPMI culture medium enriched with 10% of the athlete’s own serum and 2% of penicillin. The solution (PBMC + medium) was placed in a 6-well plate (2 ml/well) and incubated for 2 h at 37 °C for monocyte adherence. Lymphocytes were then placed in a 96-well plate (2 × 10^5^ cells/well) and stimulated or not with PMA 0,05% for 24 h. Lymphocytes medium and cells were separated by centrifugation (1800 rpm, 10 min, 4 °C) and stored at − 80 °C for further analyses.

### Immunophenotyping

One-hundred (100) µl of heparinized whole blood were added to a tube containing 2 ml of RBC Lysis Solution (Quiagem, USA), incubated at 37 °C for 10 min; the cell pellet was obtained after centrifugation (1800 rpm, 10 min, 4 °C). The pellet was washed twice (before and after centrifugation) and labelled with monoclonal antibodies against CD45 (PERcP-BL3), CD3 (FITC-BL1), CD4 (APC-RL1) and CD8 (PE-BL2), CD45Ra (Alex700-RL2) and CD27 (Pacific Blue-VL1) (Fig. 7B). The cells were incubated in the dark for 30 min, washed with PBS once and centrifuged. The final pellet was resuspended with PBS 2% BSA and then read in a flow cytometry Attune NxT (Thermo Fhisher Scientific, USA). Evaluation of lymphocyte memory subsets was only conducted 1 day before, 1 h after, and 5 days after the race.

### Cytokine quantification

Production of cytokine interleukin (IL)-2, IL-4, IL-6, IL-8, IL-10, IL-15, IL-1β, Tumor Necrosis Factor Alpha (TNFα) and Interferon-Gamma (IFNγ) in the plasma from whole blood, stimulated or not with LPS and in the medium of lymphocyte cell culture was assessed by Multiplex (R&D Systems, USA).

### RNA isolation, reverse transcription, and real-time PCR

The total RNA from lymphocytes was extracted as described by Chomczynski and Sacchi (1987)^54^ and quantified in a spectrophotometer (260 nm/280 nm). cDNA was synthesized from total RNA using reverse transcriptase performed with a high-capacity cDNA kit (Applied Biosystems, Foster, CA). The gene expression was quantified by real-time PCR using power SYBR Green Master Mix (Applied Biosystems) as fluorescent dye. GAPDH expression was used as endogenous control. The sequence of primers is shown in Supplementary Table 2.

### Statistical analyses

Data were analyzed in GRAPHPAD PRISM 5.0 . After the verification of data distribution, the data were consider as normal, so the treatment used was two-way ANOVA with multiple comparation, “group” and “time” as factors, and Tukey’s post-hoc test (*p* < 0.05). Results were expressed as means ± standard deviation (SD).

## Supplementary information

Supplementary Information
