# Supplementary material for: Probiotic supplementation in marathonists and its impact on lymphocyte population and function after a marathon: a randomized placebo-controlled double-blind study
Source: Sci Rep. 2020 Nov 2;10:18777. doi: 10.1038/s41598-020-75464-0 (PMC7608678; doi:10.1038/s41598-020-75464-0)
Supplement: Supplementary file 1 — Supplementary Information [file 41598_2020_75464_MOESM1_ESM.docx]

**Probiotic supplementation in marathonists and its impact on lymphocyte population and function after a marathon: A randomized placebo-controlled double-blind study**

Helena A.P. Batatinha1; Edgar Tavares-Silva2; Geovana S.F. Leite3; Ayane S. Resende3; José A.T. Albuquerque4; Christina Arslanian4; Ricardo A. Fock5; Antônio H. Lancha Jr3; Fabio S. Lira6; Karsten Krüger7; Ronaldo Thomatieli-Santos2,8; José C. Rosa-Neto1

1Immunometabolism Research Group, Institute of Biomedical Sciences, University of São Paulo (USP), São Paulo, SP, Brazil.

2 Department of Bioscence, Universidade Federal de São Paulo, Santos, Brazil.

3Laboratory of Applied Nutrition and Metabolism, School of Physical Education and Sports, University of São Paulo, Brazil.

4Department of Immunology, Institute of Biomedical Sciences, University of São Paulo, São Paulo, Brazil. 5Department of Clinical and Toxicological Analysis, School of Pharmaceutical Sciences, University of São Paulo, São Paulo, Brazil.

6Physical Education Department, São Paulo State University - UNESP. Brazil.

7 Department of Exercise Physiology and Sports Therapy, Justus-Liebig-University Giessen, Giessen, Germany.

^8^ Department of Bioscience, Universidade Federal de São Paulo, Santos, Brazil.

## Address for correspondence:

Helena Batatinha

Department of Cell and Developmental Biology, University of São Paulo. 1524, Prof Lineu Prestes Av. Sao Paulo, SP, Brazil, 05508-000.

+55 155 3091 0883

E-Ma[il: batatinha.helena@usp.br](mailto:batatinha.helena@usp.br)

**Supplementary material**

**Supplementary Table 1- Training volume reported by the Athletes during the supplementation period (30 days before the marathon)**

| TIME (Hours) | | | | | | | | | |
| --- | --- | --- | --- | --- | --- | --- | --- | --- | --- |
| **Week 1** | | p | | **Week 2** | p | **Week 3** | p | **Week 4** | p |
| **PLA** | 4.03±2.24 | 0.33 | 4.28±0.52 | | 0.99 | 3.33±2.08 | 0.95 | 4.11±2.08 | >0.99 |
| **PRO** | 6.30±3.33 |  | | 5.31±2.30 |  | 4.28±2.38 |  | 4.26±2.52 |  |
| DISTANCE (KM) | | | | | | | | | |
| **Week 1** | | p | | **Week 2** | p | **Week 3** | p | **Week 4** | P |
| **PLA** | 44.08±27.70 | 0,92 | | 54.90±28.58 | >0.99 | 74.48±57.25 | 0.91 | 77.48 ±85.61 | 0.75 |
| **PRO** | 69.21±29.97 |  |  | 62.67±25.49 |  | 48.28±24.76 |  | 44.10±22.15 |  |

PLA=Placebo; PRO= Probiotic

**Supplementary Table 2- Blood cells count**

|  | **Rest** | | **Pre** | **Post** | **Recovery** | **p** |
| --- | --- | --- | --- | --- | --- | --- |
| **Leucocytes**  **(****x10^3^/ul)** | **PLA** | 5.57±0.99 | 5.43±1.16 | 13.1±4.34^abc^ | 5.55±1.01 | >0.001 |
|  | **PRO** | 5.83±1.60 | 5.59±1.68 | 13.25±4.54^abc^ | 6.09±1.1 |  |
| **Neutrophils**  **(x10^3^/ul)** | **PLA** | 3.18±1.72 | 3.35±11.94 | 11.46±13.19 ^abc^ | 4.00±3.19 | >0.001 |
|  | **PRO** | 3.21±1.70 | 3.56±2.20 | 11.92±4.74^abc^ | 4.11±2.74 |  |
| **Monocytes**  **(x10^3^/ul)** | **PLA** | 0.57±0.22 | 0.47±0.09 | 0.88±0.30 ^abc^ | 0.49±0.14 | >0.001 |
|  | **PRO** | 0.53±0.16 | 0.46±0.15 | 0.90±0.25 ^abc^ | 0.54±0.15 |  |
| **Lymphocytes**  **(x10^3^/ul)** | **PLA** | 1.59±0.37 | 1.79±0.50 | 1.07±0.66 ^b^ | 1.66±0.49 | >0.001 |
|  | **PRO** | 1.78±0.46 | 1.70±0.38 | 1.08±0.30 ^abc^ | 1.83±0.55 |  |
| **Red Blood Cell**  **(x10^6^/ul)** | **PLA** | 5.07± 0.22 | 5.04±0.27 | 4.95±0.39 | 5.03±0.29 | 0.98 |
|  | **PRO** | 5.26±0.41 | 5.22±0.43 | 5.32±0.52 | 5.24±0.49 |  |
| **Hemoglobin**  **(g/dl)** | **PLA** | 15.26±0.84 | 15.06±0.80 | 14.01±0.98 | 15.27±0.76 | 0.79 |
|  | **PRO** | 15.48±0.84 | 14.92±0.63 | 15.25±1.06 | 15.07±0.84 |  |
| **Hematocrit**  **(%)** | **PLA** | 45.2±1.96 | 45.33±2.14 | 42.5±2.77 | 45.17±2.02 | 0.95 |
|  | **PRO** | 44.90±2.09 | 45.08±1.75 | 45.57±2.66 | 44.57±2.37 |  |
| **MCV**  **(FL)** | **PLA** | 89.21±3.47 | 89.87±2.97 | 85.9±3.07 | 88.83±2.48 | 0.87 |
|  | **PRO** | 85.73±6,.9 | 87.12±6.77 | 86.07±6.93 | 85.55±7.04 |  |
| **MCH**  **(pg.)** | **PLA** | 30.10±1.14 | 29.86±1.22 | 28.5±1.27 | 30.01±0.86 | 0.78 |
|  | **PRO** | 28.98±2.46 | 28.84±2.55 | 28.82±2.49 | 28.96±2.58 |  |
| **MCHC**  **(g/dl)** | **PLA** | 33.76±0.81 | 33.23±0.92 | 33.2±0.82 | 33.81±0.79 | 0.10 |
|  | **PRO** | 33.77±0.67 | 33.06±0.79 | 33.46±0.95 | 33.82±0.50 |  |
| **Plaquettes**  **(x10^3^/ul)** | **PLA** | 221.63±76.82 | 236.46±32.63 | 223±42.37 | 239.25±35.74 | 0.17 |
|  | **PRO** | 234.26±54.80 | 232±38.74 | 248.73±36.34 | 245.57±52.03 |  |

PLA=Placebo; PRO= Probiotic; MCV= Mean corpuscular volume; MCH= Mean corpuscular hemoglobin; MCHC = Mean corpuscular hemoglobin concentration ; ^a^= diferente do basal; ^b^=diferente do momento prova; ^c^= diferente do momento recuperação;

**Supplementary table 3- Primers sequence.**

| Gene | Primer Forward | Primer Reverse |
| --- | --- | --- |
| PLZF | GAGATCCTCTTCCACCGCAAT | CCGCATACAGCAGGTCATC |
| RORC | GTGGGGACAAGTCGTCTGG | AGTGCTGGCATCGGTTTCG |
| TLR-4 | TTTGCTCTTATGGATTGTCCCC | CATTGATGCAGCACAGTTGTC |
| NF-κB | GAAGCACGAATGACAGAGG | GCTTGGCGGATTAGCTCTTTT |
| GAPDH | ACAACTTTGGTATCGTGGAAGG | GCCATCACGCCACAGTTTC |

Supplementary figure 1. Total number of CD4 T memory subsets: Naïve (A); central memory (B); effector memory (C); effector memory RA (D). Marathonists supplemented with placebo (grey circle) or probiotic (black square). (two-way ANOVA followed by Tukey). (N=6 in each group)
